# Near-infrared light-responsive on-demand puerarin-releasing injectable hydrogel for promoting healing of infected wounds

**DOI:** 10.1016/j.mtbio.2025.101817

**Published:** 2025-05-05

**Authors:** Shaobo Zhai, Jiaqian You, Zheng Yang, Yang Liu, Tianming He, Yuchuan Wu, Xuyan Wei, Mucong Li, Xiaolu Shi, Fengxiang Gao, Shunli Chu

**Affiliations:** a Jilin Provincial Key Laboratory of Tooth Development and Bone Remodeling, Hospital of Stomatology, Jilin University, Changchun, 130021, Jilin, China; b Changchun Institute of Applied Chemistry, Chinese Academy of Sciences, 5652 Renmin Street, Changchun, 130021, China; c Hospital of Stomatology, Guanghua School of Stomatology, Sun Yat-sen University and Guangdong Provincial Key Laboratory of Stomatology, No.56, Lingyuan West Road, Yuexiu District, Guangzhou, 510055, China

**Keywords:** Self-healing, Injectable hydrogel, Low-temperature PTT, Immunomodulation, Wound healing

## Abstract

Wound infection is a serious clinical challenge, often accompanied by persistent inflammatory response and the threat of increased bacterial resistance, there is an urgent requirement to develop a wound dressing with potent antimicrobial as well as immunomodulatory effects. In this study, we developed a multifunctional wound dressing to promote healing of infected wounds by doping puerarin-loaded mesoporous polydopamine nanoparticles into a carboxymethyl chitosan/sodium alginate oxide-based hydrogel. The hydrogel has good injectability, adhesion, and self-healing capabilities, and can be well matched to irregular wounds and against external injuries. The loaded PUE is rapidly released from the hydrogel in response to near-infrared (NIR) laser irradiation, which has a controlled release property. In addition, under NIR laser irradiation, the hydrogel with good photothermal properties exerted good antimicrobial properties through localized high temperature, and exhibited good antioxidant, immune microenvironment modulation, and pro-angiogenic effects through accelerated released PUE. In the E. coli-infected full-thickness skin wounds healing experiments, the hydrogel can effectively kill bacteria, regulate the immune microenvironment, promote collagen deposition and angiogenesis, and significantly accelerate the wound healing, showing promising applications.

## Graphical abstract

**Figure:**
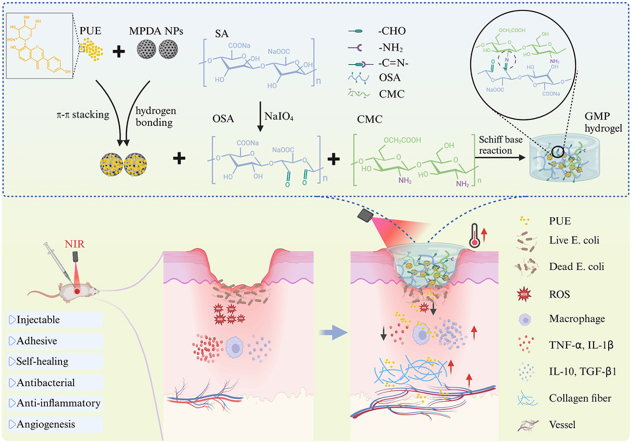

## 1 Introduction

The Skin, which covers the surface of the bodies, is an important barrier that protects the body from damage caused by external harmful substances and the environment [1,2]. The integrity of the skin may be destroyed as a result of trauma, surgery, etc., and secondary infections of wounds are one of the challenges in the treatment of trauma. The formation of a bacterial biofilm can greatly increase the difficulty of bacterial removal, which, together with persistent local inflammation, leads to delayed healing of the wound [3,4]. Some infections may even lead to serious complications such as uremia and sepsis [5]. Meanwhile, angiogenesis, as a crucial biological event driving tissue repair, is responsible for regulating the immune response and reconstructing the tissue microenvironment, which is the key link between hemostasis, inflammation, proliferation and remodeling stages, and its abnormal function will directly lead to delayed wound healing or even the formation of hard-to-heal wounds [6]. Therefore, the development of new wound dressings with efficient bacterial killing, inflammation regulation and vascular network reconstruction functions has become an urgent clinical challenge.

Ideal wound dressings need to precisely regulate the microenvironment in the four stages of hemostasis-inflammation-proliferation-remodeling [[7], [8], [9]]. Hydrogels are preferred for new generation of functional dressings due to their three-dimensional porous network mimicking extracellular matrix, excellent hydrophilicity, biocompatibility and dynamic osmotic balance [[10], [11], [12], [13], [14], [15]]. Especially, injectable self-healing hydrogels based on natural polymers have shown unique advantages due to their ability of in situ molding and matching irregular wounds [16,17]. Chitosan (CS) and sodium alginate (SA) are natural biopolymers with antimicrobial capacity, which are widely used in medical dressings due to their abundant sources and biocompatibility [18,19]. Carboxymethyl chitosan (CMCS) is a derivative of CS, which improves the disadvantage of poor water solubility of CS, and possesses amino cations that could disturb the synthesis of macromolecules on the surface of bacteria and influence the cell wall permeability, thereby exhibiting excellent hemostatic and antimicrobial abilities [[20], [21], [22], [23], [24], [25]]. The amino group of carboxymethyl chitosan (CMCS) and the aldehyde group generated by the oxidation of sodium alginate are dynamically crosslinked by the Schiff base reaction to form OSA/CMCS injectable self-healing hydrogels with dynamic imine bonding [26]. However, these materials rely on a single contact bactericidal mechanism facing two major challenges in complex infected microenvironments: (i) insufficient efficiency in removing dormant bacteria in biofilms; and (ii) lack of ability to regulate infection-induced excess reactive oxygen species (ROS) and pro-inflammatory cytokines, such as TNF-α and IL-6, making it difficult to achieve a good therapeutic efficacy in fighting infections and promoting tissue repair [[27], [28], [29]].

In recent years, near-infrared (NIR) laser-induced photothermal therapy (PTT) has been favored for its noninvasive and precisely targeted antimicrobial properties, but its clinical application is limited by two major bottlenecks: (i) high temperatures (>55 °C) can lead to damage of adjacent normal tissues, and (ii) the generation of excessive ROS during photothermal processes can exacerbate oxidative stress [30,31]. In addition, bacterial infections mobilize inflammatory cells to exert phagocytosis, this process can generate large amounts of reactive oxygen species (ROS), exacerbate oxidative stress and lead to adverse effects such as reduced collagen deposition and angiogenesis, delayed epithelial reformation, etc., which prolongs the inflammatory phase and slows wound healing [32,33]. Therefore, integrating low-temperature PTT (<50 °C) with natural ROS-scavenging drugs represents a promising strategy to synergistically eliminate pathogens while maintaining redox homeostasis. This dual-functional system not only minimizes thermal damage to healthy tissues and suppresses excessive ROS generation but also establishes a dual-protective microenvironment for infected wounds through combined "photothermal antibacterial, anti-inflammatory, and antioxidant" effects. Puerarin (PUE), is an isoflavone derivative derived from the Chinese medicine Pueraria Mirifica with a wide range of pharmacological activities such as anti-inflammatory, antimicrobial, improving microcirculation, and promoting angiogenesis, etc. [34,35]. PUE has a strong anti-inflammatory and antioxidant capacity, which can scavenge excessive ROS, inhibit lipid peroxidation and cellular damage, exerting favorable effects on wound healing. However, limitations such as low water solubility and low bioavailability have greatly restricted its application [36]. Mesoporous polydopamine (MPDA), as a biomaterial that mimics mussel adhesive proteins, is endowed with strong tissue-adhesive capacity due to its unique catechol structure, and possesses highly efficient photo-thermal conversion ability, which can disrupt the bacterial membrane structure by low-temperature photothermal therapy (45–50 °C). It has plentiful aromatic rings, which can carry insoluble drugs through π-π stacking and/or hydrogen bonding, and the porous structure of its surface can further enhance its drug-loading capacity [[37], [38], [39], [40]]. In addition, MPDA NPs can be adhered to skin tissues via Michael addition and Schiff base reactions between amino and thiol groups of skin tissues, as well as non-covalent bonding including hydrogen bonding and π-π stacking, which reduces physical loss of drug-carrying nanoparticles due to degradation of the hydrogel and further improves drug utilization [[41], [42], [43]]. Therefore, using mesoporous polydopamine as the drug-carrying platform to enhance the bioavailability of PUE and realize the synergistic effect of “bactericidal - anti-inflammatory - pro-repair” provides a safe and effective therapeutic strategy for the treatment of infected wounds.

**Scheme 1:**
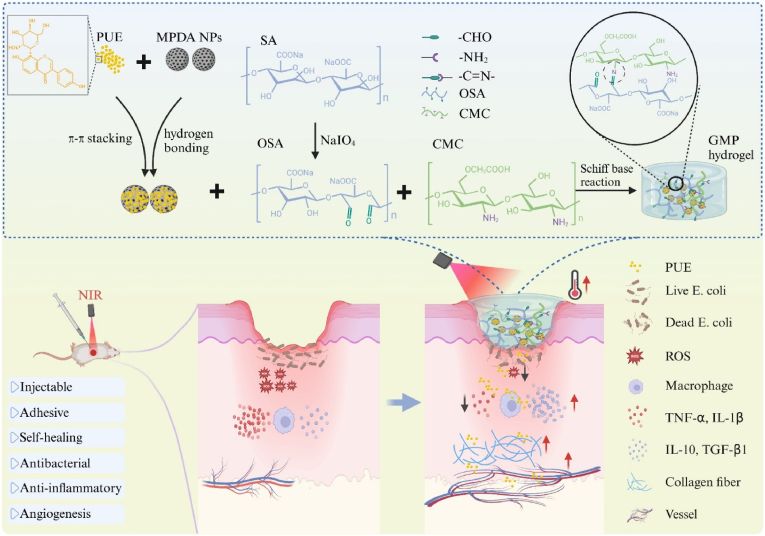
Schematic illustration of the preparation of the multifunctional GMP hydrogel and treatment in infected wounds.

In this study, we synthesized MPDA NPs as a carrier platform for PUE by one-pot method, loading PUE into MPDA NPs by π-π stacking and/or hydrogen bonding, and then doped it into CMCS and OSA polymers to prepare a nanocomposite hydrogel MPDA@PUE/CMCS/OSA hydrogel (Gel-MPDA@PUE, abbreviated as GMP hereafter) by the Schiff base reaction between CMCS and OSA (Scheme 1). It was demonstrated that the hydrogel had good injectability and self-repairing properties, which could meet the matching of irregular wounds and resist external damage. The GMP hydrogel has a good photothermal effect, which, in addition to maintaining the long-term release of PUE, exhibits controlled release properties in response to NIR laser irradiation, improving its solubility and bioavailability. In addition, the localized high temperature generated gives it a good antimicrobial effect. Meanwhile, PUE released in response to near-infrared laser radiation could remove excessive accumulated ROS, regulate immune microenvironment, and promote angiogenesis. The healing-promoting effect of GMP + NIR treatment was evaluated in an E. coli-infected mouse full-layer skin wound model. The results demonstrated that GMP hydrogel perfectly combined the antimicrobial effect of low-temperature PTT and the anti-inflammatory, antioxidant, and angiogenesis-promoting abilities of PUE under NIR light irradiation, combining the functions of scavenging infections, modulating immune microenvironment, and promoting angiogenesis and collagen deposition, making it a promising candidate for dealing with infected wounds.

## 2 Materials and methods

### 2.1 Materials

Puerarin (PUE), dopamine hydrochloride, 1,3,5-trimethylbenzene (TMB), tris(hydroxymethyl)aminomethane hydrochloride (Tris-HCl), and Pluronic F127 were obtained from Aladdin Industrial Co. (Shanghai, China). Dulbecco's modified Eagle medium (DMEM) and fetal bovine serum (FBS) were acquired from Hyclone. All reagents were utilized without further purification.

### 2.2 Synthesis of MPDA NPs

Mesoporous polydopamine nanoparticles (MPDA NPs) were prepared by one-pot synthesis [44]. The template was prepared by dissolving 360 mg PF-127 and 415 μL TMB into the mixture of 65 ml deionized water and 60 ml absolute ethanol, followed by reaction for 30 min. Subsequently, 60 mg of dopamine hydrochloride and 90 mg of Tris-HCl were added (pH = 8.5). After 24 h of reaction, MPDA NPs were collected by centrifugation (12,000 rpm min^−1^, 15 min), washed 3 times with anhydrous ethanol/acetone mixture (v/v = 2:1) and water in turn. The ultimate outcome was resuspended in deionized water.

### 2.3 Preparation of MPDA@PUE NPs

To load PUE, MPDA NPs were gently mixed with a solution of PUE dissolved in anhydrous ethanol in a ratio of 1:2. Following 4 h of stirring in the absence of light, the collected MPDA@PUE NPs was obtained through centrifugation and subsequently subjected to triple washing with deionized water. UV–vis absorption at a wavelength of 250 nm was employed to determine the loading capacity of PUE. The drug loading efficiency and encapsulation efficiency of MPDA@PUE were calculated via eqs (1), (2)), respectively:

(1) drugloadingefficiency=PUEloaded(mg)×100%/MPDA@PUENPs(mg)

(2) encapsulationefficiency=PUEloaded(mg)×100%/totalPUE(mg)

### 2.4 Synthesis of OSA

1g SA was dissolved in 100 ml of distilled water first. Thereafter, 1.08g sodium periodate was added into the dissolved SA solution. The solution was stirred for 6 h at room temperature in the dark. Then, ethylene glycol 1.5 ml was added to stop the reaction. Continually, the crude polymer was dialyzed against distilled water for 3 days with several changes of water every day. Finally, OSA was regained by freeze-drying method.

### 2.5 Preparation of hydrogels

CMCS, OSA were first dissolved in phosphate-buffered saline (PBS) to obtain CMCS solution (40 mg ml^−1^), OSA solution (160 mg ml^−1^). Then, the MPDA@PUE NPs were dispersed in OSA solution to obtain the OSA/MPDA@PUE solution. The CMCS solution and OSA/MPDA@PUE solution were thoroughly mixed and then placed at room temperature for a few minutes to form the antibacterial composite hydrogels (the mass ratio of CMCS to OSA was 3:2, and the concentration of MPDA NPs in the hydrogel was 0.5 mg ml^−1^). The CMCS/OSA hydrogel, CMCS/OSA/MPDA hydrogel and CMCS/OSA/MPDA@PUE hydrogel was named as G, GM and GMP, respectively.

## 3 Results

### 3.1 Preparation and characterization of MPDA and MPDA@PUE NPs

**Fig. 1:**
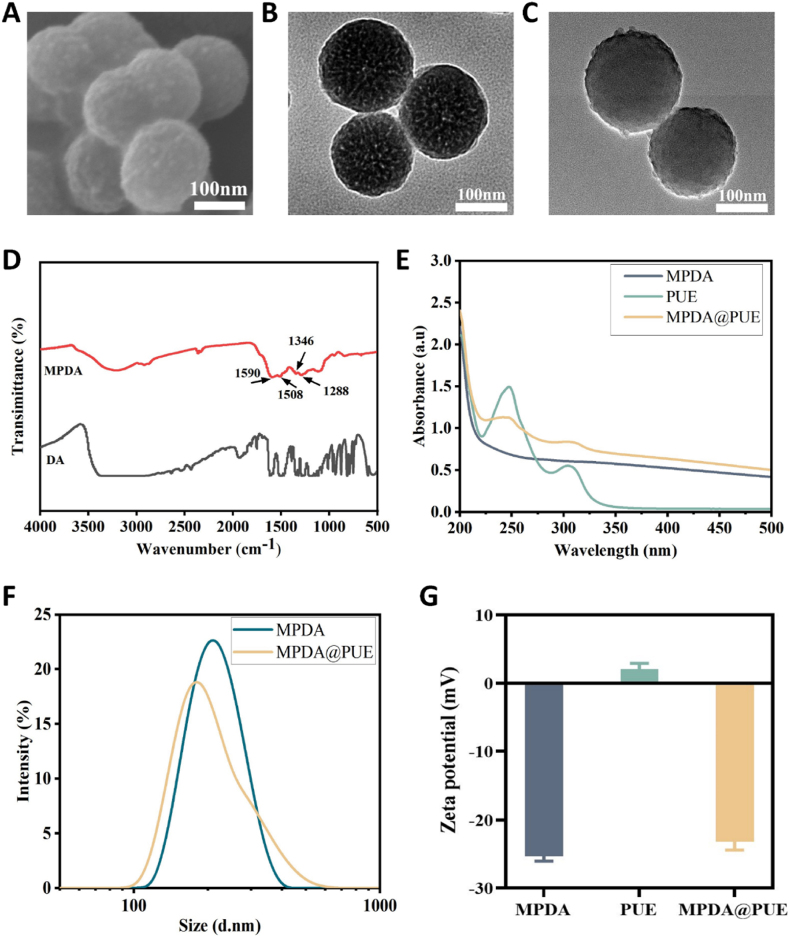
Characterization of MPDA@PUE NPs (A) SEM image and (B) TEM image of MPDA NPs. (C) TEM image of MPDA@PUE NPs. (D) Fourier transform infrared (FT-IR) spectroscopy of the DA and MPDA NPs. (E) UV–vis spectra of the MPDA NPs, MPDA@PUE NPs, and PUE. (F) DLS size distribution of MPDA and MPDA@PUE NPs. (G) Zeta potential of the MPDA NPs, MPDA@PUE NPs, and PUE.

MPDA NPs were synthesized via a self-assembly process of primary DA in an alkaline environment using pluronic F127 and 1,3,5-trimethylbenzene (TMB) as organic templates [44]. Subsequently, PUE was loaded onto the surface and within the mesopores of MPDA by π-π stacking. It can be observed by scanning electron microscopy (SEM) and transmission electron microscopy (TEM) that the synthesized MPDA NPs are in spherical shape with clearly distinguishable mesoporous structures on the surface, and their particle size is about 170 nm (Fig. 1A and B). After loading PUE, there is no obvious change in the morphology, but the mesoporous structures on the surface become blurred due to the loading of PUE into the mesoporous structures (Fig. 1C). In the FTIR spectra of MPDA NPs, characteristic peaks near 1508 and 1590 cm^−1^ could be attributed to bending vibration of N-H on the aromatic ring, and peaks near 1346 and 1288 cm^−1^ belonged to the C-O-H bending and tensile vibrations of the benzene ring (Fig. 1D). The results demonstrate the successful preparation of MPDA NPs. The UV–vis spectrum of PUE showed a characteristic UV absorption peak at 250 nm, and the MPDA@PUE NPs also showed a new absorption peak at around 250 nm (Fig. 1E). Dynamic light scattering (DLS) shows that the hydrodynamic size of MPDA is about 204.9 nm, and after loading PUE, the size increases to 253.7 nm (Fig. 1F). In addition, zeta potential measurement showed that the zeta potentials of MPDA NPs were changed from −25.4 ± 0.656 mV to −23.2 ± 1.2 mV due to the loading of positive charge PUE (Fig. 1G). These results prove the successful loading of PUE. In addition, the concentration standard curve of PUE was determined by UV spectroscopy and the drug loading efficiency and encapsulation efficiency were calculated to be about 32 % and 23.53 %, respectively (Fig. S1).

### 3.2 Fabrication of MPDA@PUE embedded CMCS/OSA hydrogel

**Fig. 2:**
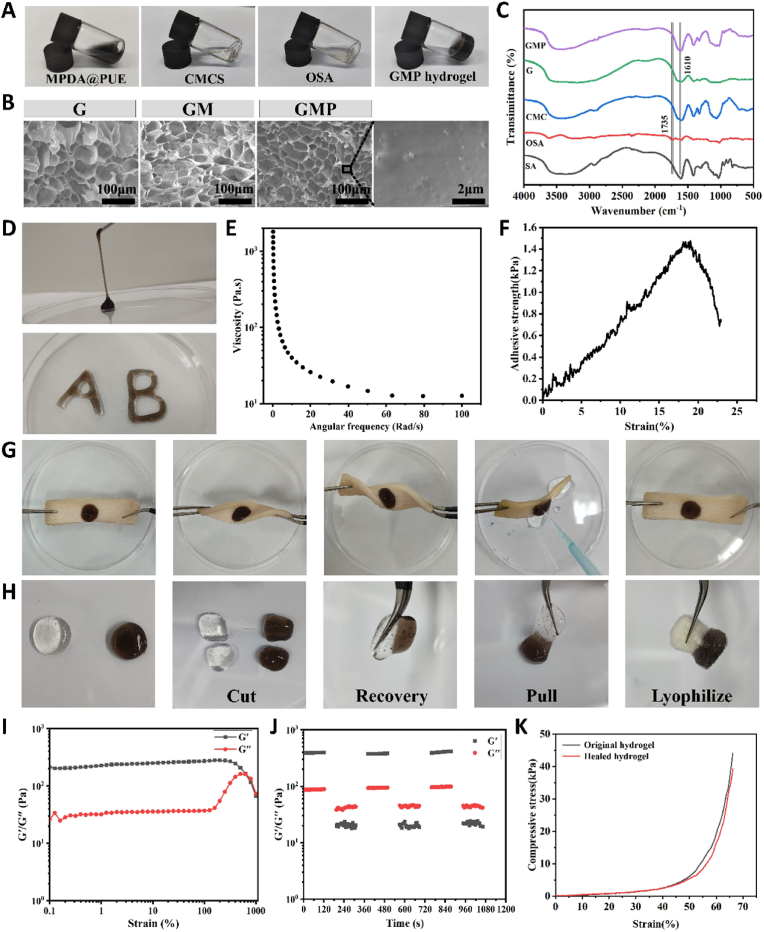
Formation and characterization of GMP hydrogel. (A) Photographs of MPDA@PUE, CMCS, OSA solution, and GMP hydrogel. (B) SEM image of the G, GM and GMP hydrogels. (C) FT-IR spectroscopy of the SA, OSA, CMCS, and different hydrogels. (D) Pictures of the injectability of GMP hydrogel. (E) Viscosity values with angular frequency variety of GMP hydrogel. (F) Lap shear test of GMP hydrogel to porcine skin. (G) Pictures of GMP hydrogel adhesion. (H) Self-healing pictures of GMP hydrogel. (I) Amplitude scan test curves of GMP hydrogel. (J) Dynamic rheological cycling test of GMP hydrogel. (K) Strain-compressive stress curves of the healed hydrogel and the initial hydrogel.

The hydrogel was prepared based on the Schiff base reaction between CMCS and OSA, and dynamic imine bonds dynamic imine bonds formed endowed the hydrogel with excellent in situ gelation properties. Based on the reaction between quinone groups of MPDA@PUE and nucleophiles like amine group and thiol group [45], MPDA@PUE can form composite hydrogels by reacting with amine groups of CMCS, which shows an obvious black color (Fig. 2A). SEM images showed that the prepared hydrogels presented a homogeneous porous structure with interconnections, which facilitated oxygen permeation and wound exudate absorption, and some MPDA@PUE NPs could be observed in the GMP hydrogels (Fig. 2B). As shown in (Fig. 2C), compared with SA, a new peak at 1735 cm^−1^ in the FT-IR spectra of OSA, attributed to the stretching vibration of the C=O bond of aldehyde group, in addition, the degree of oxidation of OSA was measured to be about 51.98 % using hydroxylamine hydrochloride method, these results prove successful synthesis of OSA. Whereas, in the FTIR spectra of each group of hydrogels, the peak at 1735 cm^−1^ of the aldehyde group disappeared and the stretching vibration absorption peak of the imine bond was shown at 1610 cm^−1^, indicating the successful synthesis of the hydrogels. The amino content of CMCS was measured to be about 2.8 mmol g^−1^ by acid-base titration method.

### 3.3 Injectable, self-healing, and adhesive properties of hydrogels

As shown in Fig. 2D, the hydrogel can be injected into the culture dish smoothly and continuously with a syringe, and it can be written “A” and “B” easily, which proves that the prepared hydrogel has good injectability. In order to understand the injectable properties of the hydrogel, the viscosity variation with angular frequency was investigated, as shown in Fig. 2E, the viscosity of the hydrogel decreases significantly with the increase of angular frequency, which suggests that it has good injectable properties and can be injected into irregular areas to fill the wounds, and it also makes it promising to be applied to minimally invasive treatment in clinics. We tested the adhesive strength of the hydrogel using the lap shear test, which demonstrated its good adhesive ability to skin tissues, and its integrity can be maintained on repeated twisting of the pig skin and did not fall off, which is favorable for its maintenance in the trauma (Fig. 2F and G). The colorless hydrogel and the black hydrogel were cut separately, and the two halves could be merged into a complete hydrogel after contact without external force, and would not be disconnected by pulling, which proved that the hydrogel had good self-healing properties (Fig. 2H). In addition, the rheological properties of GMP hydrogels were evaluated using energy storage modulus (G′) and loss modulus (G″). As shown in Fig. 2I, the amplitude scan test curve of the hydrogel shows that G′ decreases and G″ increases with the increase of strain, and the two curves intersect at 640 % strain, indicating that the hydrogel network is disrupted. Subsequently, the self-healing ability was evaluated by dynamic rheological cycling tests, which showed that at a large strain (γ = 700 %), the energy storage modulus and loss modulus were significantly inverted (G′<G″), indicating that the gel network was broken. Whereas, the hydrogel network recovered (G′>G″) when the strain was reduced to 1 %. After three cycle changes, the G′ of hydrogel did not change significantly, indicating that the hydrogel network could realize complete recovery after damage(Fig. 2J). As shown in Fig. 2K, the strain-compressive stress curves of the healed hydrogel and the initial hydrogel are almost coincident, and the compressive strength of the healed hydrogel can be restored to about 92.3 % of the initial hydrogel at 65 % strain. These results demonstrate that the prepared hydrogel has good self-healing properties. Therefore, the prepared hydrogel can well adapt to complex wounds and resist external damage.

### 3.4 Photothermal performance assay

**Fig. 3:**
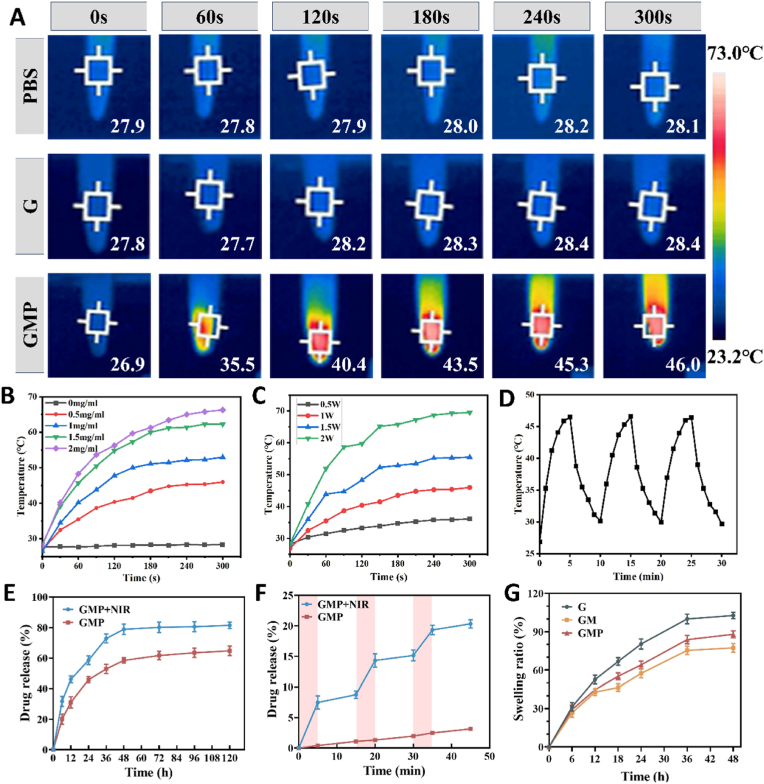
Photothermal properties of hydrogels. (A) Real-time thermal images of PBS, G, and GMP hydrogels (0.5 mg ml^−1^) under NIR (808 nm, 1.0 W cm^−2^) irradiation for 5 min. (B) Temperature change curves of hydrogels with different MPDA NPs concentration (0, 0.5, 1, 1.5 and 2 mg ml^−1^) in water under NIR irradiation (1.0 W cm^−2^). (C) Temperature change curves of GMP hydrogel (0.5 mg ml^−1^) in water under different powers of NIR irradiation (0.5, 1, 1.5, and 2 W cm^−2^). (D) Photothermal cycling test of GMP (0.5 mg ml^−1^) under 808 nm laser irradiation (1.0 W cm^−2^) for 3 laser on/off cycles. (E) Drug release profile of GMP hydrogel at 37 °C and NIR (808 nm, 1.0 W cm^−2^) irradiation. (F) PUE release behavior with three cycles under 808 nm laser irradiation (1.0 W cm^−2^, irradiated for 5 min and followed by an interval of 10 min in each cycle). (G) Swelling ratio behaviors of G, GM and GMP hydrogels.

In addition, we investigated the effects of MPDA NPs concentration and the NIR laser power intensity on the photothermal properties of the hydrogel. As shown in (Fig. 3A), the temperature of the hydrogel reached a plateau after about 4 min of 808 nm laser irradiation, whereas the temperature of the phosphate-buffered saline (PBS) and nanoparticle-free hydrogels was almost unchanged. The hydrogel with different concentrations (0, 0.5, 1.0, 1.5, and 2.0 mg ml^−1^) of MPDA NPs reached temperatures of 28.4, 46, 53, 62.3, and 66.3 °C, respectively, after 5 min of irradiation (1.0 W cm^−2^) (Fig. 3B). The temperature of hydrogels (0.5 mg ml^−1^) reached 36.1, 46.0, 55.5 and 69.5 °C after 5 min of laser irradiation with NIR laser at different laser power (0.5, 1.0, 1.5 and 2.0 W cm^−2^), respectively (Fig. 3C). In addition, the hydrogel still had high photothermal performance after three “On-Off” cycle processes with the 808 nm laser, indicating its good photothermal stability (Fig. 3D). The results show that the temperature change of the hydrogel depends on the laser power and the concentration of nanoparticles, which also proves that the prepared hydrogel has a good photothermal effect. The heat generated by the photothermal effect can enhance tissue regeneration and lead to the rupture of bacterial cell membranes and protein inactivation, but higher temperatures (∼50 °C) may also lead to the death of normal cells. And the presence of heat shock proteins can repair cell damage caused by lower temperatures such as 42–47 °C [46,47]. Therefore, for the subsequent experiments we chose a laser power intensity of 1 W cm^−2^ to exert the antimicrobial effect while avoiding damage to normal cells from excessive temperatures. In addition, NIR laser irradiation of GMP hydrogels for 5 min per day was performed to assess the drug release rate from the hydrogels, using hydrogels without NIR irradiation as a control. The results showed that NIR laser irradiation significantly increased the drug release rate, a phenomenon that could be attributed to the heat generated by the photothermal effect breaking the π-π stacking and/or hydrogen bonds between MPDA and PUE (Fig. 3E) [48]. Then, three cycles of NIR irradiation (5 min for each irradiation and 10 min intervals) were performed to evaluate the drug release. It was observed that the release of PUE increased sharply under NIR irradiation, whereas only a small amount of PUE was released without NIR irradiation (Fig. 3F). This suggests that the NIR laser effectively triggered the accelerated drug release from the hydrogel. The above results proved that the hydrogel could maintain the stable release of PUE and release it rapidly in response to NIR irradiation, achieving the controlled release of PUE. Good slow release as and controlled release of drugs is a key factor in the clinical translation of these hydrogels, which can greatly improve the bioavailability of drugs, and alleviate the adverse effects, endowing them with good prospects for clinical applications.

### 3.5 Swellability and degradation of hydrogel

Swellability is a crucial parameter for hydrogels. Good swellability allows hydrogels to absorb excess wound exudate and maintain a moist microenvironment of the wound, but excessive swelling can also have adverse effects such as reduced mechanical properties and increased risk of infection. Fig. 3G shows the swelling rate of hydrogel immersed in PBS, it can be seen that the swelling balance is reached after about 36 h of immersion and the swelling rate of nanoparticle-doped hydrogel is lower than that of nanoparticle-free group, which suggests that the interactions between the nanoparticles and the polymer chains can limit its excessive swelling [49]. In addition, we used pig skin to simulate a skin wound and injected the hydrogel into it, then placed it into PBS, and after it was sufficiently swelling, we observed that the hydrogel was still well maintained in place and did not fall off or break after undergoing repeated twisting (Fig. S2). Suggesting that the prepared hydrogel can meet its application. Fig. S3 shows the degradation rate of the hydrogels and it can be seen that the degradation rate of GM as well as GMP hydrogel groups is lower than that of G, which is favorable for the maintenance of the wound dressing on the wound surface and the sustained release of the drug. Presumably, it is due to the Schiff base and/or Michael addition reaction between the quinone groups of MPDA NPs and the amine groups of CMCS as well as between the amine groups of MPDA NPs and the aldehyde groups of the OSA increasing the cross-linking density of the hydrogel network [50,51]. At the same time, the appropriate degradation rate also provides the possibility of its future application *in vivo*.

### 3.6 Biocompatibility and intracellular ROS tests of hydrogels

**Fig. 4:**
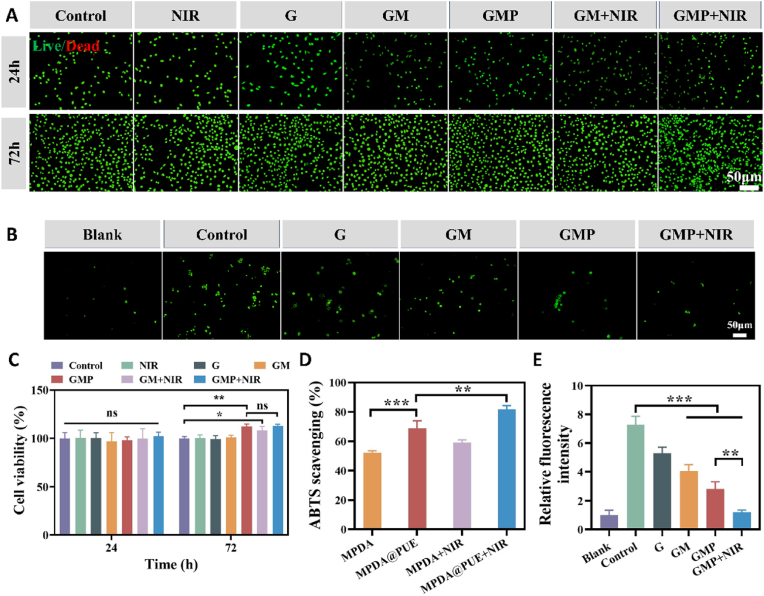
Biocompatibility and antioxidant properties of hydrogels. (A) Fluorescent images of live/dead L929 cells cocultured with different hydrogels for 24 and 72 h. (B) ROS scavenging ability of hydrogels. (C) Cell viability of L929 cells after cocultivation with the hydrogels by the CCK-8 assay. (D) ABTS scavenging ability of MPDA and MPDA@PUE NPs under NIR irradiation. (E) Quantitative analysis of the ROS scavenging ability of different hydrogels. (n = 3, ∗p < 0.05, ∗∗p < 0.01, and ∗∗∗p < 0.001; “ns” denoted no significant difference, error bars indicate means ± standard deviations).

As a wound dressing, the biocompatibility of hydrogel is crucial, so the biocompatibility of hydrogel was evaluated by CCK-8 analysis and live/dead cell staining.CCK-8 analysis showed that the hydrogel did not show cytotoxicity to mouse fibroblasts (L929), and the survival rate was more than 90 % in all groups, and the nanoparticle-doped hydrogel showed the effect of promoting cell growth after 3 day, indicating that the hydrogels had no adverse effect on the long-term survival of the cells. The cells in all groups showed good cell viability, most of the cells in all groups were viable (green), few dead cells were observed (red), and the cells in all groups had good cell morphology or structure (Fig. 4A and C). This indicates that the prepared hydrogels have good biocompatibility.

Excessive oxidative stress resulting from excessive ROS accumulation leads to metabolic disorders, lipid peroxidation, cellular senescence and death, and even damage of tissues, and scavenging of excessive ROS facilitates wound healing [52,53]. The extracellular antioxidant capacity of the hydrogel was evaluated by its ability to scavenge azidobis (3- thylbenzothiazoline-6-sulfonic acid) (ABTS) free radicals. Considering that PTT may induce a localized photothermal effect leading to elevated ROS, we explored the effect of NIR radiation on the free radical scavenging efficiency of nanoparticles by ABTS radials scavenging tests. The results demonstrated the outstanding free radical scavenging ability of MPDA@PUE NPs under NIR laser irradiation (Fig. 4D). This finding highlights their efficacy as antioxidants after PTT treatment. Subsequently, we measured the intracellular ROS levels in RAW264.7 cells using 2,7-dichlorofluorescein (DCFH)-DA probe to further evaluate the antioxidant capacity of the hydrogel. As shown in (Fig. 4B and E), strong green fluorescence (DCFH-DA) was detected by fluorescence microscopy in the control group, demonstrating the success of intracellular ROS induction. The ROS content was reduced in all hydrogel groups, while the lowest fluorescence intensity was detected in the cells of the GMP + NIR group, which was almost the same as that of the uninduced blank group, and was consistent with the results observed in the extracellular experiments, which may be due to the fact that the NIR radiation increased the release of the drug, and therefore exerted a better antioxidant effect. It was demonstrated that the prepared hydrogel can effectively scavenge intracellular ROS and has excellent extracellular and intracellular antioxidant capacity.

### 3.7 Antiinflammatory activity of hydrogels

**Fig. 5:**
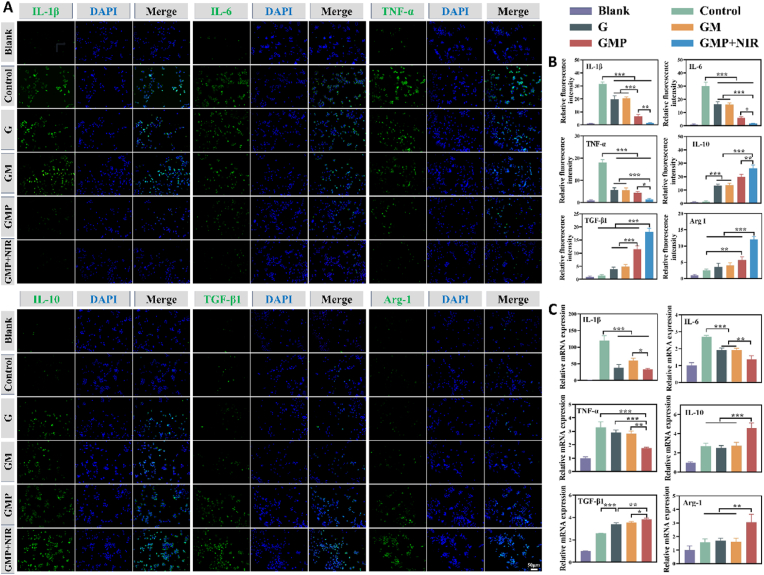
*In vitro* anti-inflammatory capacity of hydrogels. (A) Immunofluorescent staining (IF) of inflammatory factors (IL-1β, IL-6, TNF-α, IL-10, TGF-β1, and Arg-1) expressed by RAW 264.7 cells. (B) Quantification of the inflammatory factors based on fluorescence intensities. (C) Real-time PCR analysis of the mRNA expression of proinflammatory factors (IL-1β, IL-6 and TNF-α) and antiinflammatory factors (IL-10, TGF-β1 and Arg-1). (n = 3, ∗p < 0.05, ∗∗p < 0.01, and ∗∗∗p < 0.001; “ns” denoted no significant difference, error bars indicate means ± standard deviations).

We studied the anti-inflammatory capacity of hydrogels by RT-qPCR, immunofluorescence (IF) staining, respectively. Lipopolysaccharide (LPS) can bind to macrophage receptors and induce the expression of pro-inflammatory cytokines such as IL-1β, IL-6 and TNF-α. This promotes the polarization of M1-type macrophages [54,55]. We established an inflammatory microenvironment by treating RAW264.7 cells with Escherichia coli LPS, and then observed the inflammatory factors, and related mRNA expression after being treated with hydrogel. Immunofluorescence staining results showed high expression of pro-inflammatory cytokines IL-1β, IL-6 and TNF-α in the control group, and MPDA@PUE NPs-doped hydrogel significantly inhibited the expression of these inflammatory factors. In addition, the hydrogel significantly promoted the expression of anti-inflammatory related factors such as IL-10, Arg-1 and TGF-β1 compared to LPS stimulated cells. The effect was particularly significant in the GMP + NIR group, probably due to the NIR radiation promoting the release of PUE (Fig. 5A and B). In addition, RT-qPCR results showed a similar trend (Fig. 5C). A series of experiments confirmed the excellent anti-inflammatory effect of the prepared hydrogel.

### 3.8 Antibacterial activity of hydrogels

**Fig. 6:**
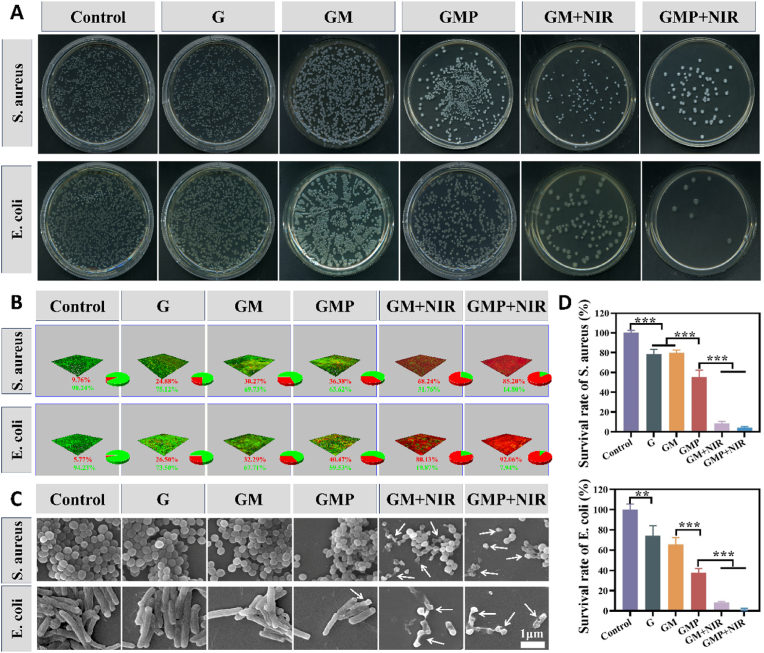
*In vitro* antimicrobial capacity of hydrogels. (A) Typical photographs of S. aureus and E. coli colonies of biofilm on agar plates with different treatments. (B) 3D live/dead staining images of S. aureus and E. coli biofilms after different treatments. The green and/or red portion of the pie chart represents the proportion of live or dead bacteria. (C) SEM images of S. aureus and E. coli biofilm; the white arrows represent bacterial cell membranes damage and degeneration. (D) Survival rate of E. coli and S. aureus. (n = 3, ∗p < 0.05, ∗∗p < 0.01, and ∗∗∗p < 0.001; “ns” denoted no significant difference, error bars indicate means ± standard deviations). (For interpretation of the references to color in this figure legend, the reader is referred to the Web version of this article.)

In order to assess the antibacterial ability of the hydrogels, we performed agar plate tests with Escherichia coli (Gram-negative bacteria) and Staphylococcus aureus (Gram-positive bacteria), which are widely present in infected wounds. As shown in Fig. 6A and D, due to the antibacterial ability of CMCS, the survival rate of bacteria in G group also showed a certain decrease, while the GM + NIR as well as GMP + NIR groups showed the strongest antimicrobial ability, the survival rate of bacteria in both of them was less than 10 %, which shows that the antimicrobial ability of the hydrogel mainly comes from the heat generated by its photothermal effect [56]. The antibacterial effect of GMP on E. coli was better than that of S. aureus, and its bacterial survival rate was 37.81 % and 55.31 %, respectively, probably because the cell wall of E. coli was thinner than that of S. aureus, and thus the resistance to drugs was lower than that of S. aureus [57]. In addition, PUE was able to bind to LPS and exert a bactericidal function similar to that of antimicrobial peptides, preventing the repair of cell membranes damaged by the photothermal effect and conferring a very high antibacterial rate against Gram-negative bacteria [58]. Three-dimensional reconstruction of live/dead fluorescence staining of bacterial biofilms further demonstrated the antibacterial effect of drug and PTT synergistic treatment on bacterial biofilms (Fig. 6B). Subsequently, the antimicrobial effects of the different treatments were qualitatively assessed by SEM, for S. aureus, it was seen that the bacterial surface showed obvious depression, crumpling, and even rupture in the group irradiated by NIR light, indicating that NIR light irradiation could effectively destroy the bacterial cell membranes. In contrast the bacteria in the control group presented a more intact morphology. Similar results were also obtained in E. coli (Fig. 6C). The results demonstrate the excellent ability of the hydrogel in antibacterial properties.

### 3.9 Cell migration and angiogenesis

**Fig. 7:**
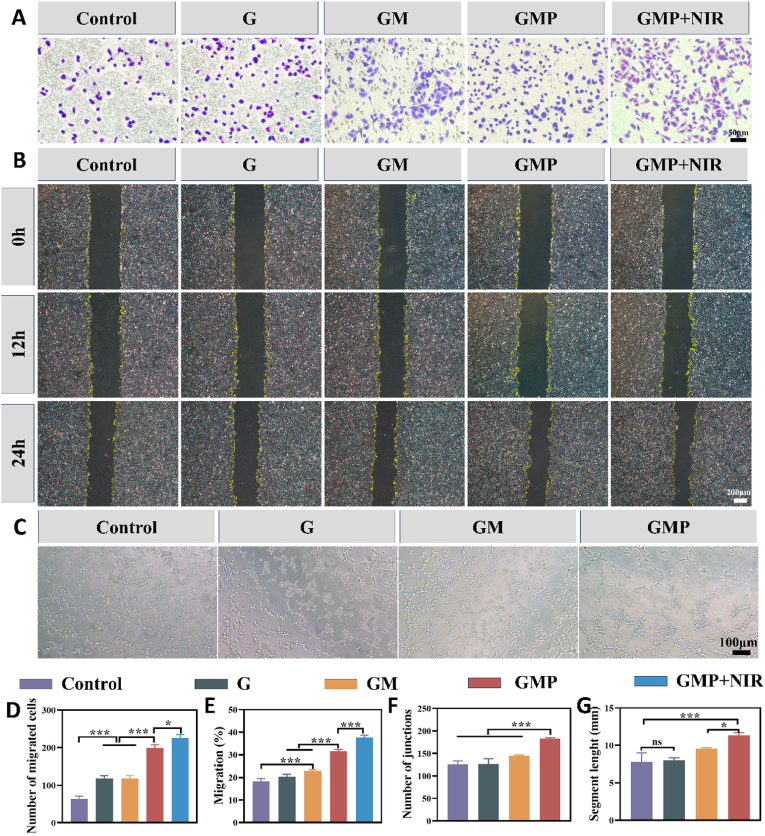
Angiogenesis assay of HUVECs. (A) Images of transwell assay. (B) Images of the scratch experiment. (C) Images of the tube formation assay. (D) Quantitative analysis of transwell assay. (E) Quantitative analysis of scratch experiments. (F) Quantitative analysis of junction. (G) Quantitative analysis of segment length. (n = 3, ∗p < 0.05, ∗∗p < 0.01, and ∗∗∗p < 0.001; “ns” denoted no significant difference, error bars indicate means ± standard deviations).

**Fig. 8:**
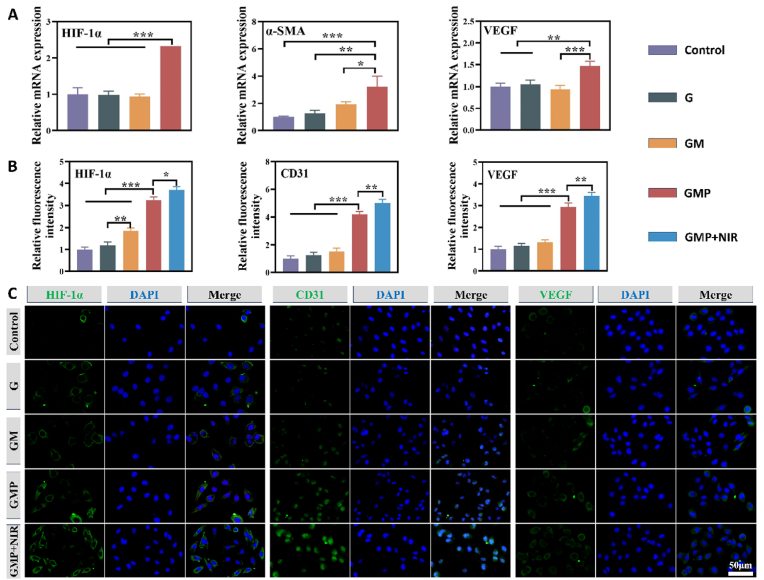
Evaluation angiogenic effect of hydrogels *in vitro* using HUVECs. (A) Real-time PCR analysis of the mRNA expression of HIf-1α, α-SMA, and VEGF. (B) Quantitative analysis of the HIF-1α, CD31 and VEGF based on fluorescence intensities. (C) IF images of angiogenesis-related proteins (HIF-1α, CD31 and VEGF) expressed HUVECs after different treatments. (n = 3, ∗p < 0.05, ∗∗p < 0.01, and ∗∗∗p < 0.001; “ns” denoted no significant difference, error bars indicate means ± standard deviations).

Cell migration and micro-angiogenesis are essential for wound healing, so an ideal hydrogel dressing should be able to promote cell migration as well as angiogenesis. Studies have shown that PUE has effective angiogenic properties, which is important for promoting tissue regeneration [59,60]. To evaluate the *in vitro* angiogenic ability of the hydrogel, we performed vascular endothelial cell scratch assay, migration assay, and tube-like structure formation assay. The results of the transwell assay showed that GMP and GMP + NIR treatments significantly promoted the migration of HUVECs (Fig. 7A and D). Meanwhile, the scratch assay also verified that the highest level of cell migration was observed in the GMP group and the GMP + NIR groups (Fig. 7B and E). These results indicated that PUE could induce the migration of HUVECs, suggesting its potential to induce angiogenesis. We subsequently used Matrigel to simulate the extracellular matrix to assess the *in vitro* tube formation behavior of each group of HUVECs. Briefly, cells were inoculated on the surface of the Matrigel and incubated with extracts of G, GM, and GMP hydrogels. The GMP group was observed to have the highest number of capillary-like networks, and its number of tubule-forming junctions and length of tubule formation were much higher than that of the other groups, demonstrating a significantly enhanced tube-forming capacity (Fig. 7C, F and G). It was demonstrated that the PUE-containing hydrogel had better angiogenic ability. Next, we investigated the regulatory effects of hydrogels on the function of HUVECs by immunofluorescence staining and RT-qPCR. HIF-1α, α-SMA, and VEGF are important factors in inducing angiogenesis. It has been shown that PUE can exert a pro-angiogenic effect by inducing the expression and activation of genes such as VEGF and HIF-1α [61,62]. To investigate the mechanism of angiogenesis induced by GMP hydrogel, the expression level of mRNA of factors related to HUVECs were evaluated. The results showed that the gene expression of HIF-1α, α-SMA and VEGF was significantly higher in the GMP group than other groups (Fig. 8A). Immunofluorescence staining results also showed that the levels of HIF-1α, VEGF and CD31 proteins were significantly higher in the GMP + NIR as well as the GMP group than in the other groups (Fig. 8B and C). Angiogenesis plays a crucial role in tissue repair and wound healing, so the combination treatment of GMP hydrogel with NIR holds great promise in promoting the healing of infected wounds.

### 3.10 Wound-healing evaluation and histopathological evaluation *in vivo*

**Fig. 9:**
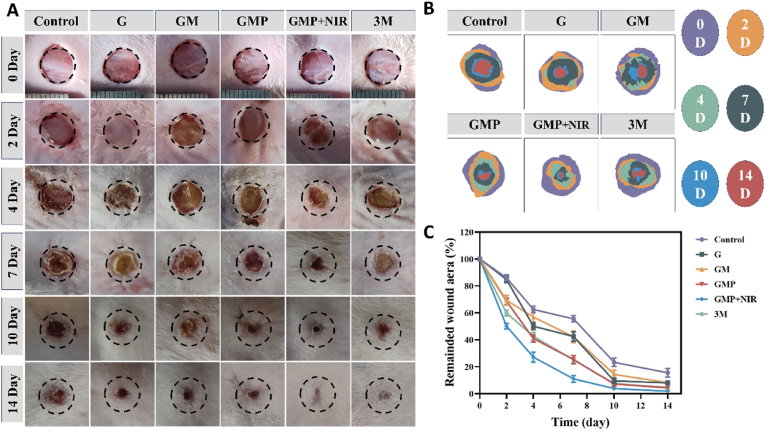
Assessment of the effectiveness of GMP hydrogels in promoting healing of infected wounds *in vivo*. (A)Representative photographs of skin wounds in each group at different time points. (B) Simulation diagram of skin wound healing for individual groups. (C) Representative changes of wound area over time.

E. coli is one of the most common Gram-negative bacteria that can cause a variety of diseases, and it is one of the five most common bacteria that cause wound infections [63,64]. We inoculated E. coli on full-thickness-skin wounds on the back of mice to establish an infected model to evaluate the effect of prepared hydrogel under NIR laser irradiation on the infected wound healing process. After exposure to NIR laser irradiation, the temperature of the wound site in the GMP + NIR group increased dramatically to about 47.5 °C, a temperature sufficient to kill the surrounding bacteria, and the temperature change around the wound emphasized its effective local PTT effect. We recorded typical images of the wound site on days 0, 2, 4, 7, 10, and 14 (Fig. 9A and B). Wound healing was assessed in each group based on the ratio of the remaining area of the wound to the area of the original wound. As shown in (Fig. 9C), the GMP + NIR group presented the highest rate of wound healing at all time points due to the excellent anti-inflammatory and angiogenic capacity, combined with the superior antimicrobial capacity conferred by the NIR laser irradiation. On day 14, the infected wounds almost completely subsided under the synergistic effect of the drug and PTT. In contrast, the control group treated with PBS and the G group presented a lower rate of healing and larger wounds at the same time points.

**Fig. 10:**
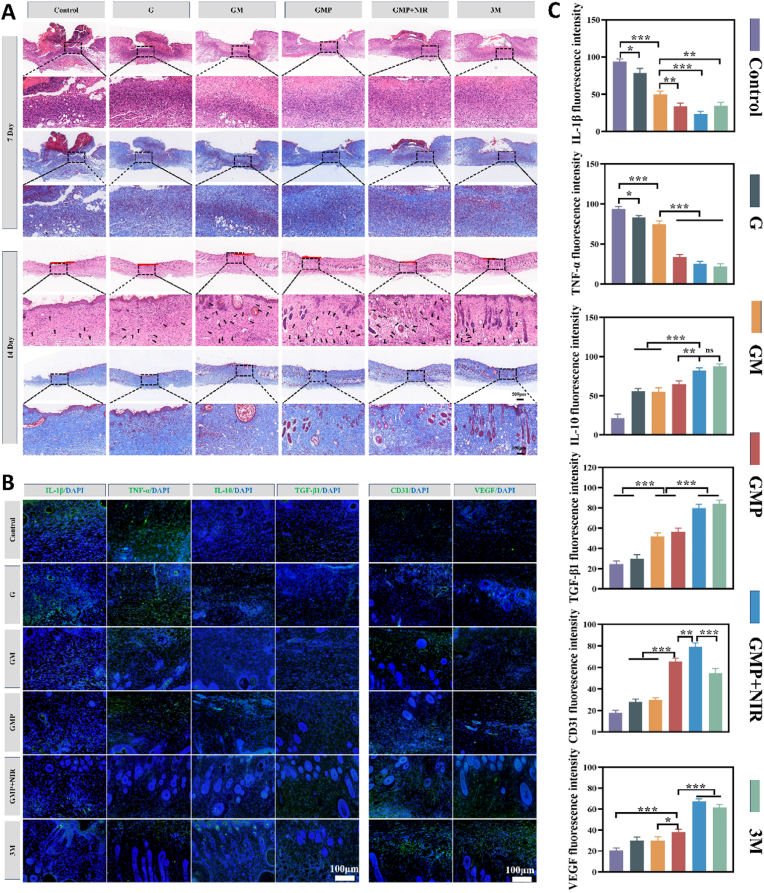
*In vivo* demonstration of GMP hydrogel promoting infected skin tissue healing. (A) Representative H&E and Masson's trichrome staining images of tissue sections collected from wound areas at day 7 and day 14. (B) Immunofluorescence images of IL-1β, TNF-α, IL-10, TGF-β1, CD31 and VEGF expressed in wounds. (C) Quantitative data of the IL-1β, TNF-α, IL-10, TGF-β1, CD31 and VEGF fluorescence intensity. (n = 3, ∗p < 0.05, ∗∗p < 0.01, and ∗∗∗p < 0.001; “ns” denoted no significant difference, error bars indicate means ± standard deviations).

Wound healing is a complex interaction between hemostasis, inflammation, proliferation of cells and remodeling of tissues. The formation of a wound scab, composed of platelets and erythrocytes, as well as fibrin, is a sign of the beginning of wound healing, and the scab is gradually absorbed and removed with wound healing. Bacterial infection can lead to inflammation and even cellular necrosis, delaying wound healing [65,66]. It was clearly observed that the wound area in the GMP + NIR group was smaller than the other groups, and the results of hematoxylin and eosin (H&E) staining also showed that there were fewer inflammatory cells around the wound than in the other groups, which proved the synergistic antioxidant/anti-inflammatory effect of the PTT treatment combined with PUE (Fig. 10A). In addition, more angiogenesis (shown by black arrows), which is essential for tissue repair, was also observed in the GMP + NIR group (Fig. 10A and S4). During wound healing, collagen deposition has an essential role in evaluating the efficiency of healing [66]. On day 14, Masson trichrome staining showed that there were more well-arranged collagen fibers deposited in the GMP + NIR group, which indicated its good healing effect (Fig. 10A and S5). IF of the tissues also showed the expression of inflammation-related factors TNF-α and IL-1β was down-regulated in the GMP + NIR group, while the anti-inflammatory factors TGF-β1 and IL-10 were obviously up-regulated, which was due to the bactericidal effect produced by the photothermal effect and the release of PUE reducing the inflammatory response, and the fluorescent signals of vascular-related proteins CD31 and VEGF in this group were significantly stronger than the other groups (Fig. 10B and C). It indicates that the PUE released from this hydrogel can well promote the various processes of healing of wounds, such as inflammation, proliferation of cells, and remodeling of tissues. In the inflammation stage, it can play a good anti-inflammatory and antioxidant role as a scavenger of ROS, and in the proliferation and remodeling stage, it also plays a favorable role in promoting fibroblast proliferation, the establishment of vascular network, and collagen deposition. In addition, H&E staining of vital organs (heart, liver, spleen, lungs, and kidneys) of the animal model did not reveal any histologic or morphological differences between each experimental group and the control group (Fig. S6). It indicates that the prepared hydrogel dressing has a good biosafety. These results suggest that GMP hydrogels with NIR irradiation are promising candidates for use in promoting healing of infected wounds.

## 4 Discussion

Currently, the treatment of infected wounds faces some key challenges, such as the fact that conventional dressings are monofunctional, only having the roles of hemostasis, exudate absorption, and providing an isolation barrier, however, they still encounter major hurdles in terms of antimicrobial activity, immunomodulation, and angiogenesis, and drug-loaded dressings often suffer from poor wound match, easy damage, short service life, low drug loading efficiency, and burst release of drugs [67]. In order to address this clinical challenge, in this study, MPDA NPs were synthesized as the drug carrier platform for PUE and doped into CMCS/OSA to prepare a multifunctional hydrogel that combines the functions of removing infections, regulating the immune microenvironment, and promoting angiogenesis, and is easy to use with good injectability and self-repairing properties.

Reconstruction of the vascular network plays a crucial role in skin wound healing, and the novel microvascular system provides oxygen and nutrient support to the nascent tissue, which is the basis for functional tissue regeneration [6]. However, most of the studies on wound dressings have focused on their antimicrobial, antioxidant and immunomodulatory effects, but less on the reconstruction of vascular network. Besides the strong antioxidant and immunomodulatory abilities, PUE also has excellent angiogenic properties, which is an important reason for us to choose it as a therapeutic drug. In order to improve the limitations of its poor water solubility and low bioavailability, we chose to use MPDA NPs as its drug carrier platform. In addition to its good biocompatibility, excellent photothermal properties, and high drug loading capacity, excellent adhesive property is the most fascinating characteristic, which allows it to adhere to the surface of any substrate. The functional groups on the surface give it tissue-adhesive properties under both wet and dry conditions, and it is highly stable under physiological conditions and does not dissolve in biological fluids [41,42]. MPDA NPs can adhere to skin tissues via Michael addition and Schiff base reactions with amino and thiol groups in skin tissues, as well as non-covalent bonding including hydrogen bonding, π-π stacking [68]. During the degradation of hydrogels, MPDA NPs can reduce the physical loss by adhering to skin tissues, to exert a longer-lasting effect, which is a significant advantage. In addition, the MPDA NPs showed a large improvement in drug loading capacity compared to the conventional nanocarrier system, obtaining a drug loading efficiency of about 32 %, and exhibited a good controlled release of PUE through the NIR-responsive mechanism, which greatly improved the drug utilization. In previous studies, PUE has been mostly used for the treatment of myocardial infarction, glaucoma, acute cerebral ischemia, and diabetes mellitus etc., but it is less used for the preparation of wound dressings (Table S3). In the present study, it was used for the treatment of infected wounds, and the drug-loaded hydrogel obtained good anti-inflammatory, antioxidant and angiogenic effects, which exerted a good effect on wound healing. The strong antioxidant and anti-inflammatory properties of this hydrogel make it suitable not only for the treatment of skin wounds but also has the potential to be used in various inflammatory diseases such as osteoarthritis and ulcerative colitis. Angiogenesis is widely involved in a variety of physiological and pathological processes, and good pro-angiogenic ability makes hydrogels makes hydrogels possibly have broader applications. He et al. prepared an injectable bioactive hydrogel for minimally invasive treatment of bone defects incorporating PUE and CS with mesoporous silica nanoparticles, which showed that PUE played a good role in alleviating oxidative stress, modulation of immune response, promotion of angiogenesis, inhibition of osteoclast differentiation, and promotion of osteoblast differentiation [69]. You et al. also demonstrated that mild thermal stimulation was beneficial in recruiting osteoprogenitors at the site of bone defects to enhance bone regeneration [70]. Therefore, it is hypothesized that the GMP hydrogel can be attempted to be used to promote bone tissue regeneration. In addition, through its good photothermal effect, the hydrogel has excellent performance in antimicrobial, the survival rate of bacteria in both E. coli and S. aureus treated with it was less than 10 %. This non-discriminatory bactericidal therapy is effective in reducing the development of bacterial resistance and offers a promising option for the treatment of various bacterial infection diseases in the clinic.

Of course, there are some limitations in this study, including that the study only tested antibacterial effects against S. aureus and E. coli in terms of antimicrobial capacity, and only selected E. coli-infected wounds for the study in the animal test, while the species infecting the wounds are usually complex, so broader antimicrobial tests should be conducted against other pathogens in subsequent studies; the mouse skin wound model used may not fully replicate human wounds and lacks long-term safety and efficacy data, so more and longer-term preclinical trials are needed to validate its application.

In addition, this study also hopes to promote the innovation in the technical field in the following aspects: (i) It is expected that the photothermal response mechanism of PDA will provide a new idea for the design of nano drug-carrying systems, and it is also hoped that smart biomaterials with more response mechanisms can be designed according to the changes of local microenvironments, such as pH and ROS, so as to realize the precise controlled release of drugs, and to promote the development of precision medicine from the field of tumor to the field of infection. It is also hoped that the material can be further endowed with intelligent monitoring functions (e.g. pH/ROS response fluorescent label), to realize the integration of diagnosis and treatment, and to promote the transition of wound management from passive treatment to active intervention. (ii) Through the dynamic cross-linking between CMCS and OSA and the design of self-healing hydrogels, it is hoped to inspire the development of the next-generation injectable and self-healing materials, which can be applied to the individualized treatment of complex wounds as well as tissue defects. (iii) It is expected to promote the application of synergistic therapy of vascular network reconstruction with antimicrobial therapies and immunomodulation, providing multi-targeted combined strategy for the treatment of infected wounds, replacing the traditional monotherapy mode.

## 5 Conclusions

In conclusion, in this study we successfully developed an injectable antimicrobial GMP hydrogel for promoting healing of infected wounds. Due to its dynamic imine bonding, the hydrogel exhibits good injectability and self-healing properties, ensuring a better match with the wound and resistance to external injury. The doped MPDA@PUE NPs can exhibit good controlled release behavior in response to NIR laser radiation. Under NIR irradiation, the hydrogel exhibits excellent antimicrobial capacity against Gram-positive (S. aureus) and Gram-negative (E. coli) bacteria through the photothermal effect of MPDA@PUE NPs and the accelerated release of PUE. In addition, the GMP hydrogel exhibited a strong antioxidant effect that could modulate the inflammatory response by scavenging excessive free radicals without significant cytotoxicity, and the hydrogel also exhibited a good capacity to promote angiogenesis due to the release of PUE. In the E. coli infected full-thickness skin defect model, the GMP hydrogel under NIR laser irradiation plays a good role in all stages of wound healing through good photothermal effect as well as modulation of inflammation, promoting collagen deposition and angiogenesis, which effectively promotes the healing of infected wounds. Therefore, it can be concluded that this study provides an idea for the design of a simple, effective and safe antimicrobial hydrogel for promoting healing of infected wounds.

## CRediT authorship contribution statement

**Shaobo Zhai:** Writing – original draft, Methodology, Data curation, Conceptualization. **Jiaqian You:** Writing – original draft, Methodology, Data curation, Conceptualization. **Zheng Yang:** Investigation, Data curation. **Yang Liu:** Investigation, Data curation. **Tianming He:** Investigation, Data curation. **Yuchuan Wu:** Investigation, Data curation. **Xuyan Wei:** Visualization, Software. **Mucong Li:** Visualization, Software. **Xiaolu Shi:** Visualization, Software. **Fengxiang Gao:** Methodology, Formal analysis, Conceptualization. **Shunli Chu:** Supervision, Project administration, Funding acquisition, Conceptualization.

## Ethics approval and consent to participate

All animal assays in this work were endorsed by the Ethics Committee of Medical Experiment Animals in the College of Basic Medicine of Jilin University (China); (the ethics approval number is 2023494).

## Consent for publication

Not applicable.

## Declaration of competing interest

The authors declare no conflict of interest.

## Data Availability

Data will be made available on request.
