# Supplementary material for: Near-infrared light-responsive on-demand puerarin-releasing injectable hydrogel for promoting healing of infected wounds
Source: Mater Today Bio. 2025 May 5;32:101817. doi: 10.1016/j.mtbio.2025.101817 (PMC12137178; doi:10.1016/j.mtbio.2025.101817)
Supplement: Multimedia component 1 [file mmc1.docx]

**Near-infrared light-responsive on-demand puerarin-releasing injectable hydrogel for promoting healing of infected wounds**

Shaobo Zhai^1^†, Jiaqian You^1,3^†, Zheng Yang^1^, Yang Liu^1^, Tianming He^1^, Yuchuan Wu^1^, Xuyan Wei ^1^, Mucong Li ^1^, Xiaolu Shi^1^, Fengxiang Gao^2^*, Shunli Chu^1^*

1 Jilin Provincial Key Laboratory of Tooth Development and Bone Remodeling, Hospital of Stomatology, Jilin University, Changchun, 130021, Jilin, China

2 Changchun Institute of Applied Chemistry, Chinese Academy of Sciences, 5652 Renmin Street, Changchun 130021, China

3 Hospital of Stomatology, Guanghua School of Stomatology, Sun Yat-sen University and Guangdong Provincial Key Laboratory of Stomatology, No.56, Lingyuan West Road, Yuexiu District, Guangzhou 510055, China

* Corresponding author

[gfx26@ciac.ac.cn](mailto:gfx26@ciac.ac.cn) (Fengxiang Gao)

[chusl@jlu.edu.cn](mailto:chusl@jlu.edu.cn) (Shunli Chu)

MATERIALS AND METHODS

1.1 Characterization of MPDA NPs, MPDA@PUE NPs, and Hydrogels

Dynamic hydrodynamic diameter (DLS) and zeta potential were analyzed by a nano Zetasizer (Malvern ZEN3600 Zetasizer Nano-ZS, Britain). Ultraviolet-visible (UV-vis) absorption spectra were recorded by dispersing the nanoparticles in an aqueous solution (BIO-TEK SYNERGY HT, USA). The different groups of the samples were analyzed by potassium bromide tablets on the FT-IR spectrometry with the range of 400–4000 cm^–1^ (FT-IR, VERTEX 80 V, Bruker, Germany). The surface morphology of the MPDA, MPDA@PUE, and hydrogels was observed using transmission electron microscopy (TEM, JEM2100F, Japan) and scanning electron microscopy (SEM, FlexSEM 1000, Hitachi, Japan) with gold sprayed.

In order to calculate the degree of oxidation, the aldehyde group content of OSA was determined using the hydroxylamine hydrochloride titration/sodium hydroxide method. 8.69 g of hydroxylamine hydrochloride was dissolved in 75 ml of deionized water, and 3 ml of 0.05% methyl orange solution was added; the above solution was diluted to 500 ml with deionized water, and the pH value of the solution was adjusted to 4.0 with NaOH; 40 mg of OSA was dissolved in 10 ml of the above solution, and it was stirred at room temperature for 5 h. Then titrate with 0.1 M standard NaOH solution until the color of the solution changes from red to yellow, and record the consumption of NaOH as *ΔV*. The formula is as follow:

Degree of oxidation = *ΔV* × *C* × 198 × 10^-3^ / 2*W* × 100%

where *ΔV* was the volume of consumed NaOH standard solution, in ml; *C* was the molar concentration of NaOH standard solution, in mol/L; *W* was the mass of the OSA sample, in g; and 198 was the relative molecular mass of the OSA monomer unit, in g/mol.

The amino content in CMCS was assessed by acid-base titration method, 0.1 g of dry sample was dissolved in 20 ml of 0.1 M HCl solution and stirred at room temperature to dissolve it completely, then methyl orange and phenolphthalein indicators were dropped into the sample respectively. Then titrate with 0.1M standard NaOH solution. When the color of methyl orange changed, the consumption of NaOH was recorded as *V*_1_ (ml), and when the color of phenolphthalein changed, the consumption of NaOH was recorded as *V*_2_ (ml). The formula is as follows:

Amino group content (mmol/g) = (*V*_2_ - *V*_1_) × *C* / *W*

where *C* was the molar concentration of NaOH standard solution, in mol/L; *W* was the mass of the CMCS sample, in g.

1.2 Photothermal Effect Determination.

The hydrogels were immersed in PBS, placed in Eppendorf tubes, and then irradiated with an NIR laser at 808 nm (1.0 W cm^-2^) for 5 min. The images and corresponding temperature variations were captured using an infrared camera.

1.3 *In Vitro* Release Studies

To investigate the release of PUE under different conditions, GMP hydrogel was placed in a dialysis bag filled with PBS, the bag was placed in a container to be dialyzed, and the amount of PUE remaining within the fluid was measured at the corresponding time points. The remnant PUE drug content in the supernatant can be detected by a UV–vis spectrophotometer at a wavelength of 250 nm. The standard curve was utilized to determine the release of PUE.

1.4 Swelling Ratio Evaluation

To determine the swelling ratios of the different samples, the formed and wet hydrogel samples were immersed in PBS and weighed after removing excess moisture from the surface using filter papers at each predetermined time interval. *W_0_* and *W_t_* are the initial weight and weight at the corresponding point time of the hydrogels, respectively. The swelling ratios of the hydrogels were calculated with the following formula:

SR = (*Wt - W_0_*) / *W_0_* × 100%

1.5 *In Vitro* Degradation Assessment

The residual weight of the various hydrogel samples was assessed by incubating them in PBS supplemented with 10% fetal bovine serum (FBS) at 37 °C. When reaching the pre-designed durations, the remaining hydrogel was rinsed with PBS, all the superficial water gently removed and the hydrogel samples lyophilized and weighted. The weight of the initial hydrogel samples were recorded as *W_0_*, and the weights at the various time points were recorded as *W_t_*. The degradation of different samples was calculated based on the following formula:

Weight loss = (*W_0_ - W_t_*) */ W_0_* × 100%

1.6 Self-healing Assay, Injection, and Adhesion Test.

All rheological tests were conducted on a rheometer (Waters Corporation, China). The strain amplitude scanning method (γ from 0.01 to 1000%) was used to record the values from the critical strain region. Self-healing tests were then performed using GMP hydrogels, and alternating strain scanning tests were conducted at a fixed angular frequency (1 rad s^-1^). Amplitude oscillatory strain was switched from small strain (γ = 1.0%) to large strain (γ = 700%) for three cycles. To study the viscoelastic behavior of the hydrogels, the frequency sweep test was performed, which covered a range of frequencies from 0.1 to 100 rad/s at strain of γ = 1%.

The adhesive strength of the hydrogel was evaluated by lap shear test. The hydrogel precursor was coated on the dorsal area of the porcine skin (20 mm × 20 mm). Subsequently, another porcine skin was covered on the other side of the gel. The adhesive strength was tested using an electronic universal testing machine at a tensile speed of 1 mm/min. The original hydrogel and the healed hydrogel with a diameter of 12 mm and a height of 9 mm were placed in an electronic universal testing machine for compressive strength testing at a compression speed of 1mm/min and the compressive strength at 65% strain was used to evaluate the healing efficiency of the hydrogel.

In the macroscopic self-healing experiment, two pieces of hydrogels were stained with differently colored dyes and cut into two-halves. The cut interfaces of the hydrogels were then brought into contact with each other at room temperature for 20 min, and the self-healing effect of hydrogels was photographed and recorded. To test the injectability of hydrogels, we loaded the hydrogels into a syringe to produce a specific shape. The hydrogel was placed on twisted pigskin to observe its adhesion ability.

1. *In Vitro* Study

2.1 Cytocompatibility Evaluation.

Mouse fibroblast cells (L929) was cultured in DMEM supplemented with 10% FBS and 1% penicillin−streptomycin (100 U ml^−1^ penicillin and 100 g ml^−1^ streptomycin) and incubated at 37 °C under an atmosphere of 5% CO_2_ and 100% humidity. Cells were inoculated into 24-well plates at a density of 2 × 10^4^ cells per well. Then, each group of hydrogels was cocultured with the cells. Cells without hydrogel served as controls, and the

complete medium supplemented with CCK-8 without cells was set as blank group. Cell viability was assessed using the CCK-8 kit in accordance with the manufacturer’s instructions. Briefly, after washing the cells three times with PBS at selected time points (days 1 and 3), 300μL of medium containing 10% (v/v) CCK-8 solution (without FBS) was added to each well, and incubation was conducted for 60 min. Then, the medium (100μL) in each well was transferred to a new 96-well plate, and the absorbance was measured at 450 nm using an enzyme marker. The viabilities of the L929 cells can be calculated

based on the following formula:

Cell viability = (OD_treated_ - OD_blank_) / (OD_control_ - OD_blank_) × 100%

To better observe the cell growth, we incubated the groups of L929 cells cultured for 3 days with live/dead reagent (Calcein AM/PI) at 37 °C for 30 min. Fluorescence images were captured using a fluorescence microscope (Olympus, Japan).

2.2 Extracellular and Intracellular Antioxidant Assay

MPDA and MPDA@PUE NPs (or Trolox) were added to ABTS and maintained at 37 °C for 1 h. An enzyme-labeled meter was used to record the reading at an optical density (OD) of 734 nm, and the ability of the material to scavenge ABTS free radicals was calculated.

RAW264.7 cells were inoculated in a 24-well plate at a density of 3 × 10^4^ cells well^-1^ for 24 h, stimulated with 1.5 mM H_2_O_2_ for 1 h, and then treated with the hydrogels for 24 h. 2,7-Dichloro-fluorescein diacetate (DCFH-DA) was incubated for 30 min, then washed with PBS three times, and then observed and imaged using a fluorescence microscope to detect the intracellular ROS level. RAW264.7 cells cultured in DMEM with and without H_2_O_2_ were used as the control group and blank group

2.3 Evaluation of *In Vitro* Anti-inflammatory Capability

LPS was selected as an irritant of a potential inflammatory state to simulate skin infection. RAW264.7 cells were inoculated in six-well plates at a density of 3 × 10^5^ well^-1^ and stimulated using LPS (1μg L^-1^) for 4 h. The cells were then cocultured with hydrogels for 24 h. RAW264.7 cells cultured in DMEM with and without LPS were used as the control group and blank group.

2.3.1 Immunofluorescence Staining Analysis.

The expression of IL-1β, IL-6, TNF-α, IL-10, Arg-1 and TGF-β1 in the protein level was redetected by Immunofluorescence Staining (IF) to evaluate the polarization of macrophages. Cells were fixed with 4% paraformaldehyde. We used anti-IL-1β primary antibody, anti-IL-6 primary antibody, anti-TNF-α primary antibody, anti-IL-10 primary antibody, anti-Arg-1 primary antibody and anti-TGF-β1 primary antibody for 4 °C overnight incubation. IgG secondary antibodies coupled with antimouse Alexa 488 were utilized for staining at room temperature for 1 h. DAPI staining was performed for 6 min. Photographs were taken by using microscopic observation and analyzed using ImageJ.

2.3.2 RT-PCR Assay of Inflammation-related Genes.

qPCR was used to detect RNA (mRNA expression levels of factors associated with the M1 and M2 phenotypes in RAW264.7 cells. The primers are listed in Table S1. RNA was extracted using Trizol (Life TecN/hPDAologies, USA), and cDNA was prepared by reverse transcription according to the manufacturer’s instructions. Gene (IL-1β, IL-6 and TNF-α) and M2 phenotype (IL-10, Arg-1 and TGF-β1) expression was assayed using the SYBR Premix Ex TaqII reagent and a QPCR Mx3005P system. The relative expression levels of different genes were calculated using the 2^-∆∆Ct^ method, and glyceraldehyde-3-phosphate dehydrogenase (GAPDH) was used as an internal reference gene. The CT values of the blank group were used as calibrators.

1. *In Vitro* Antibacterial Activity Evaluation

E. coli, S. aureus, and MRSA were cultured in an incubator at 37 °C in LB broth.

3.1 CFU Assay.

First, the bacterial solution (1 ml, 10^6^ CFU ml^−1^) was incubated with the hydrogels for 48 h at 37 °C in a temperature-controlled environment. Subsequently, the mixtures were irradiated by using an 808 nm laser (1 W cm^−2^). Afterward, bacteria were collected from the glass climbing sections. The resulting suspension containing bacteria was then diluted. Finally, the suspension was spread onto agar plates and incubated at 37 °C for 24 h. The colony-forming units (CFU) present on the different plates were counted.

3.2 Live/Dead Staining

The biofilms were stained simultaneously with SYTO and PI dyes in the absence of light for a duration of 20 min. Ultimately, a confocal laser scanning microscope was utilized to acquire 3D biofilm images, which were subsequently subjected to analysis using ImageJ software.

3.3 Morphology Observation of Bacteria in Biofilms by SEM

The specific details of culturing the bacteria were consistent with the plate-counting method described. First, the biofilm had been fixed with 2.5% glutaraldehyde. The fixed sample underwent dehydration using a series of ethanol solutions and was observed by SEM.

1. *In Vitro* Angiogenesis Analysis

Human umbilical vein endothelial cells (HUVECs) were cultured with DMED containing 10% FBS, and 1% penicillin/streptomycin in an incubator of 5% CO_2_ at 37 ℃ and passaged every 4 days.

4.1 Scratch Assay

For the wound healing experiment, 2 ml HUVECs suspension (25×10^4^/ml) were seeded on a six-well plate for 24 h. After 100% complete attachment of cells, a straight line without cells was created by a sterile 200 μL pipette tip in the middle of the well. The cell debris should be removed by washing the cells with PBS. Next, the cells corresponding well were co-culture with different hydrogel with FBS-free culturation, after which images were captured under an inverted phase microscope (OLYMPUS, Japan) at 0, 12, 24 h. Three fields were randomly selected to be calculated. The migration rate was measured using ImageJ software to calculate the scratch area after cell migration.

4.2 Transwell Assay

For the transwell assay, HUVECs were seeds on the upper chamber with FBS-free culture medium at a density of 3×10^4^ using transwell cell culture inserts of which pore size is 8mm. On the lower chamber were added corresponding hydrogels of different groups with 600 µl of culture medium. After 24 h of incubation, HUVEC migrated from the upper chamber to the lower chamber as stimulated by different stimulation, and then the samples were collected and swabbed to remove non-migrating cells remaining on the upper surface of the filter membrane. Then 4% paraformaldehyde was used to fix the cells migrating to the lower chamber for 15 minutes and stained with crystal violet (Beyotime) for 10 min finally. The quantitative analysis of migrated HUVECs in five randomly selected fields was calculated by ImageJ software. The above experiment was repeated 3 times.

4.3 Tube Formation Assay

The tube formation assay was performed to measure the effect of different groups on the ability of HUVECs to form blood tubes, using Matrigel basement membrane matrix as the substrate material. Pre-cool the 48-well plate and pipette tips at -20 °C in the refrigerator, then allow the Matrigel to defrost for 24 hours at 4 °C. Place the 48-well plate on an ice box and add 100 μL of Matrigel to each Well. after confirming that there were no bubbles, the well plates were placed in a cell culture incubator for 0.5 hours to allow the matrix gel to solidify. Next, the digested cells were resuspended in the extract solution of different hydrogels, and then the cells were seeded onto the matrigel at a density of 3×10^4^ per well. HUVECs were cultured for 5-8 h, after which the tube formations were recorded in the field of view under the microscope (Olympus, Japan) randomly. Three random fields were selected for the calculation of the number of junctions and the segment length using ImageJ software.

4.4 IF Analysis.

For the IF Staining of HIF-1α, CD31, and VEGF staining, HUVECs were co-culture with different groups for 3 days and then were fixed by 4 % paraformaldehyde for 15 min respectively, followed by being washing by PBS three times, incubated in Immunol staining blocking buffer (Beyotime, Shanghai, China) for 1 h. Then cells were incubated with a specific primary antibody: HIF-1α Rabbit Monoclonal Antibody, CD31 Rabbit Monoclonal Antibody and VEGF Rabbit Monoclonal Antibody (diluted with Primary Antibody Dilution Buffer) overnight at 4 °C, and treated with Alexa Fluor 488-labeled Goat Anti-rabbit IgG (Beyotime, Shanghai, China) for 1h at room temperature. Finally, the images were captured by fluorescence microscopy. The nuclei were subsequently stained with DAPI (Solarbio, China) for 10 min respectively. All the above stained images were captured with an inverted microscope (Olympus, Japan). The immunostaining fluorescence intensity of cells was evaluated by ImageJ software. The experiments were repeated three times.

4.5 RT-PCR Assay

The effects of the different groups on the expression of vascularization-related genes of HUVECs including VEGF, HIF-1α, and α-SMA were evaluated through RT-qPCR. HUVECs were co-cultured with different groups for 3 days at a density of 40×10^4^ per well in the 6-well plate. Cellular RNA of HUVECs was extracted by TRIzol (TAKARA, Kusatsu, Japan) and reverse-transcribed by TAKARA Reverse Transcriptase kit (TAKARA, Osaka, Japan) as indicated of instructions. The quality and concentration of RNA were calculated by Thermo NANODROP 2000c (Thermo Fisher Scientific, Fremont, CA). Finally, the polymerase chain reaction (PCR) was performed through PrimeScript™ RT-PCR kit (TAKARA, Tokyo, Japan) and Applied Biosystems 7300 (ThermoScientific, Waltham, MA) followed by the instructions. The gene primers are shown in Table S2. The data which was normalized to the expression of GAPDH was analyzed through the 2^-∆∆Ct^ method. Each experiment was repeated three times.

1. Mouse Cutaneous Wound Infection Model and Wound-Healing Test *in Vivo*.

All animal assays in this work were endorsed by the Ethics Committee of Medical Experiment Animals in the College of Basic Medicine of Jilin University (China); (the ethics approval number is 2023494). Overall, 36 ICR mice (6-8 weeks old, 18-22 g, male) were randomized into 6 groups. After the mice were anesthetized, the backs of the mice were shaved and disinfected. Full-thickness circular skin wounds with a diameter of 8 mm were taken, and each wound was inoculated with a E. coli suspension (50μL, 1 ×10^8^ CFU ml^-1^). Bacterial infections were then treated *in vivo* using hydrogels, and infected controls were treated with PBS. Aquacel Ag (3M) was used as a positive control group. The wound that had been treated underwent a 5 min session of laser treatment using an 808 nm laser at a power density of 1 W cm^-2^. After 7 and 14 days of treatment, all mice were executed and peripheral wound tissues were collected and fixed in paraformaldehyde (4%) for further histological analysis. Subsequently, the above samples were subjected to H&E staining and Masson trichrome staining.

Immunofluorescent Staining of Biomarkers in Wounds.

First, we used paraffin-embedded soft tissue sections. Then, the above sections were stained with primary antibody at 4 °C overnight. Finally, the sections were incubated with a secondary antibody at room temperature for 60 min, and the nuclei were stained with DAPI at room temperature for 10 min. All images were captured on a fluorescence microscope (Olympus, Japan).

1. Statistical Analysis.

All experiments were performed in at least three replicates. Statistical analysis was performed using GraphPad Prism 9.5.1, and the significance was determined using a one-way or two-way analysis of variance. Statistically significant differences are denoted as *p < 0.05, **p < 0.01, ***p < 0.001, “ns” denoted no significant difference.

**Table S1.** The usage of RT-qRCT primers for inflammation-related genens.

| Genes | Sequences | |
| --- | --- | --- |
| GAPDH | F: 5’-TGACCACAGTCCATGCCATC-3’ | R: 5’-GACGGACACATTGGGGGTAG-3’ |
| IL-1β | F: 5’-TGCCACCTTTTGACAGTGATG-3’ | R: 5’-TGATGTGCTGCTGCGAGATT-3’ |
| IL-6 | F: 5’-CACTTCACAAGTCGGAGGCT-3’ | R: 5’-TCTGACAGTGCATCATCGCT-3’ |
| TNF-α | F: 5’-CAGGCGGTGCCTATGTCTC-3’ | R: 5’-CGATCACCCCGAAGTTCAGTAG-3’ |
| IL-10 | F: 5’-ATGCTGCCTGCTCTTACTGACTG-3’ | R: 5’-ATGCTGCCTGCTCTTACTGACTG-3’ |
| Arg-1 | F: 5’-TGTGTCCAGGCTCCAAATATAG-3’ | R: 5’-AGCAGGTAGCTGAAGGTCTC-3’ |
| TGF-β1 | F: 5’-CTAAGGCTCGCCAGTCCCC-3’ | F: 5’-CTAAGGCTCGCCAGTCCCC-3’ |

**Table S2.** The usage of RT-qRCT primers for angiogenesis-related genens.

| Genes | Sequences |  |
| --- | --- | --- |
| GAPDH | F: 5’ - AGAAGGCTGGGGCTCATTTG - 3’ | R: 5’ - AGGGGCCATCAGTCTTC - 3’ |
| VEGF | F: 5’ - CAGAAGGAGGAGGGCAGAA - 3’ | R: 5’ - GTCTCGATTGGATGGCAGTAG - 3’ |
| HIF-α | F: 5’ - AGAAACCACCTATGACCTGCT - 3’ | R: 5’ - CGACTGAGGAAAGTCTTGCTA - 3’ |
| α-SMA | F: 5’ - GACGACGAATCTTCTCAATGG - 3’ | R: 5’ - TGTGTCAGTTTACGATGGCAG - 3’ |


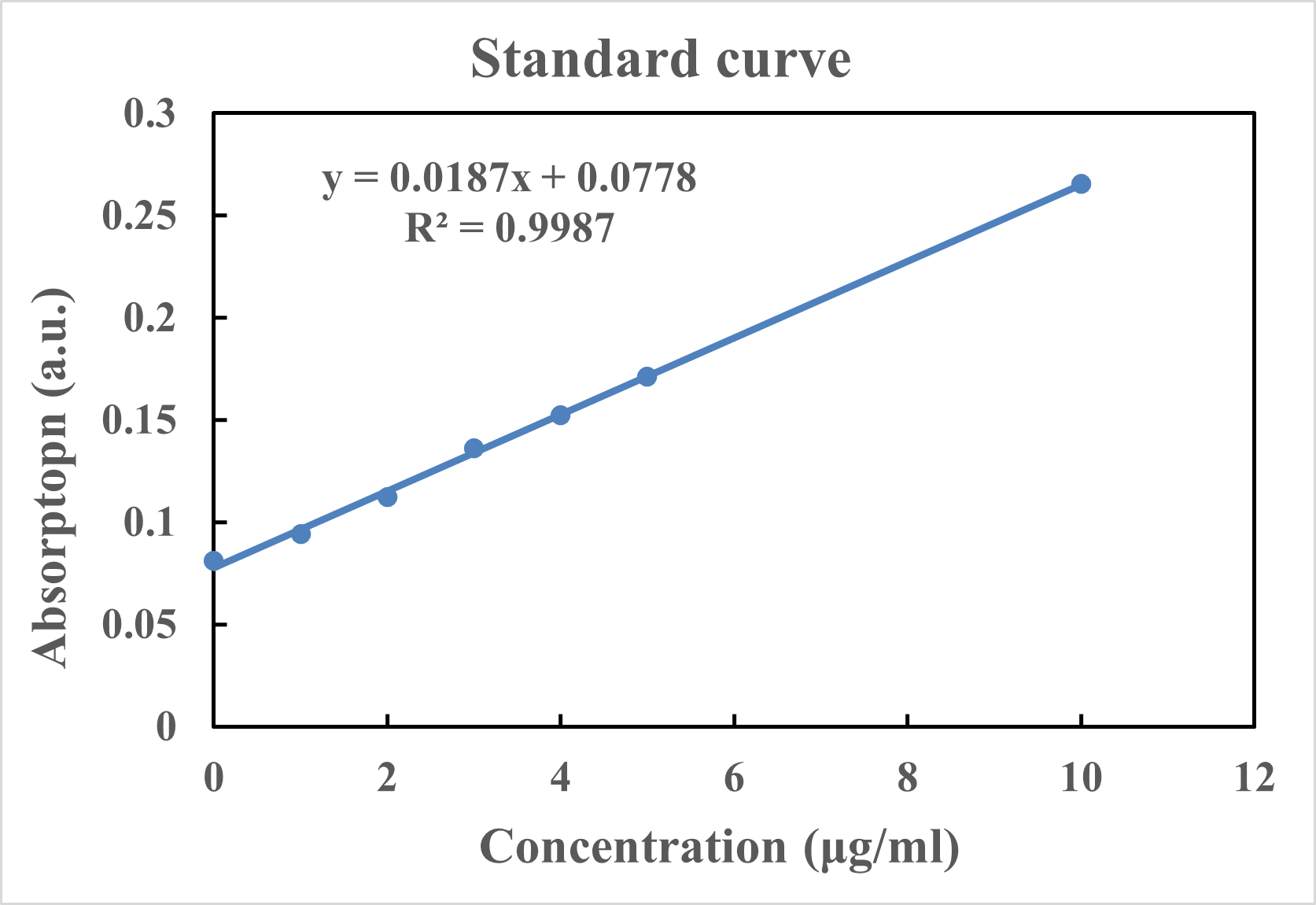


**Figure S1.** The standard curve for PUE solutions was detected at 250 nm by a UV–vis.

**Figure S2. Pictures of swollen hydrogel adhering to pigskin wounds.**


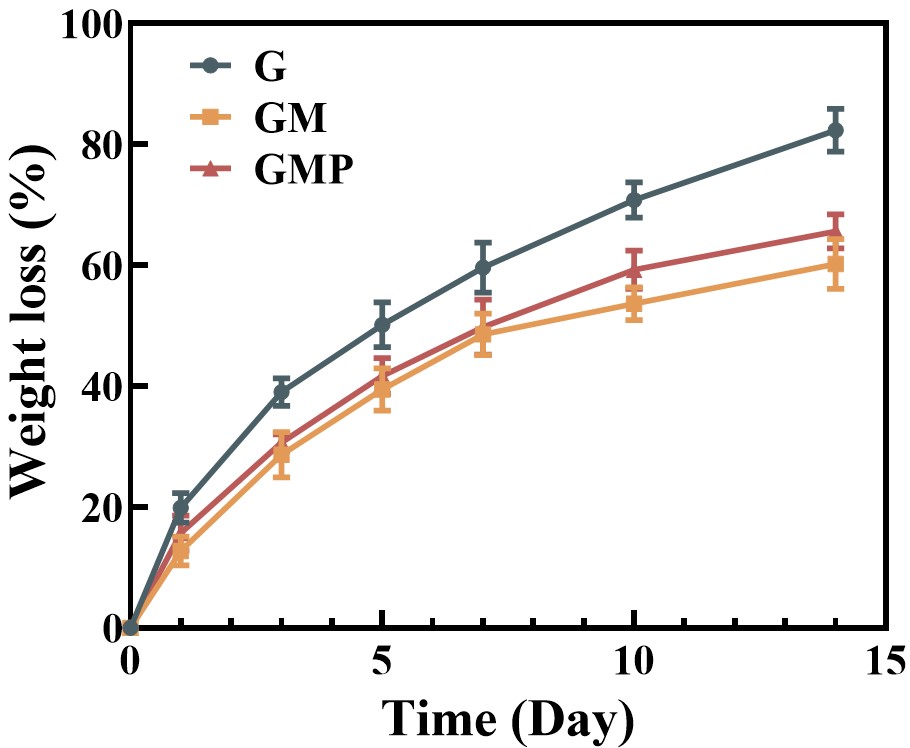


**Figure S3.** Degradation behaviors of G, GM and GMP hydrogels.


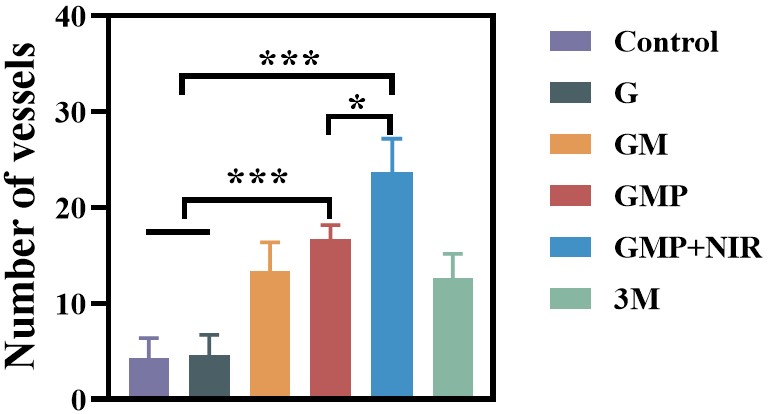


**Figure S4.** Quantitative data of the number of vessels. (**P* < 0.05, ***P* < 0.01, and ****P* < 0.001)


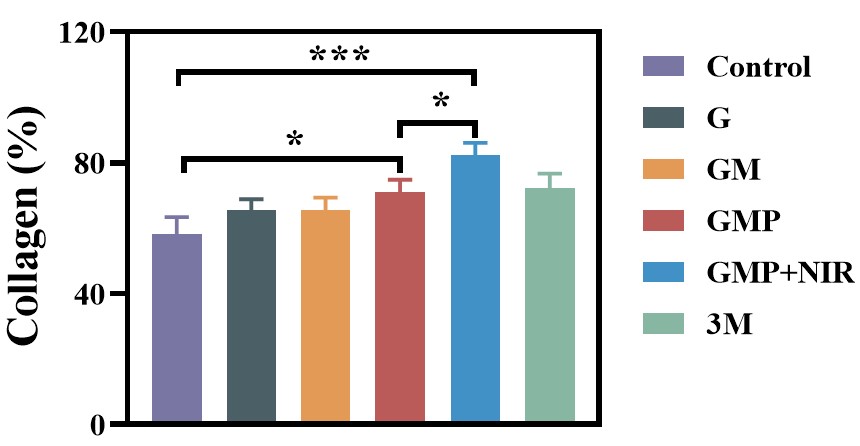


**Figure S5.** Quantitative data of the number of collagen fibers deposited. (**P* < 0.05, ***P* < 0.01, and ****P* < 0.001)


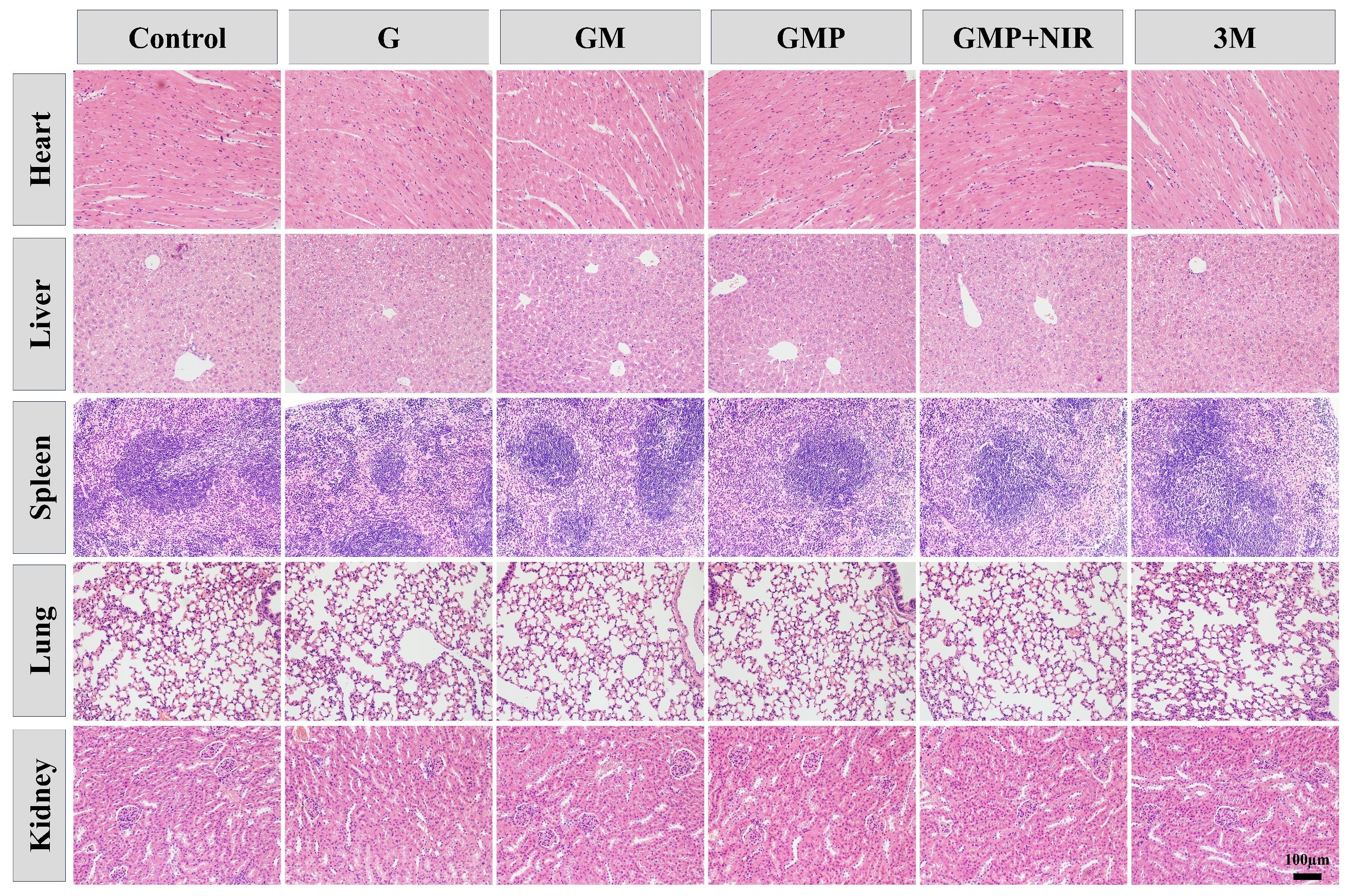


**Figure S6.** H&E staining images of major organs harvested from various groups of mice 14 days after treatment.

**Table S3.** **Application of PUE-loaded nanoparticle drug delivery system**

| Carrier system | Drug loading efficiency | Application | References |
| --- | --- | --- | --- |
| PLGA NPs | 15.50% | Ischemia–reperfusion injury in spinal cord | [1] |
| Collagen NPs | 2.68% | Ischemic stroke | [2] |
| Solid lipid NPs | 16.5% | Acute myocardial infarction | [3] |
| Albumin NPs | 7.1% | Glaucoma | [4] |
| MgO-Based PDA NPs | 7.93% | Parkinson's disease | [5] |
| MSNs NPs | 19.75% | Alcoholic liver disease | [6] |
| MSN-TPGS NPs | 13.61% | Ulcerative colitis | [7] |
| Se NPs | 10.64% | diabetes mellitus | [8] |
| Solid lipid NPs | 17.6% | Myocardial infarction | [9] |

**REFERENCES**

[1] W. Chen, Z. Zhao, S. Zhao, L. Zhang, Q. Song, Resveratrol and Puerarin loaded polymeric nanoparticles to enhance the chemotherapeutic efficacy in spinal cord injury, Biomedical microdevices 22(4) (2020) 69.

[2] G. Xu, C. Ma, H. Chu, W. Hu, L. Yang, S. Li, Anti-Inflammatory Combination of Puerarin and Ac2-26 Using Intranasal Delivery for Effective Against Ischemic Stroke in Rat Model, International journal of nanomedicine 20 (2025) 3825-3842.

[3] Z. Dong, J. Guo, X. Xing, X. Zhang, Y. Du, Q. Lu, RGD modified and PEGylated lipid nanoparticles loaded with puerarin: Formulation, characterization and protective effects on acute myocardial ischemia model, Biomedicine & pharmacotherapy = Biomedecine & pharmacotherapie 89 (2017) 297-304.

[4] L. Hu, Y. Xu, H. Meng, Development and Evaluation of Puerarin Loaded-Albumin Nanoparticles Thermoresponsive in situ Gel for Ophthalmic Delivery, Drug design, development and therapy 16 (2022) 3315-3326.

[5] Y. Gao, Y. Cheng, J. Chen, D. Lin, C. Liu, L.K. Zhang, L. Yin, R. Yang, Y.Q. Guan, NIR-Assisted MgO-Based Polydopamine Nanoparticles for Targeted Treatment of Parkinson's Disease through the Blood-Brain Barrier, Advanced healthcare materials 11(23) (2022) e2201655.

[6] X.X. Zhang, Y.F. Lang, X. Li, Z. Li, Y.Q. Xu, H.Q. Chu, The protective effect of puerarin-loaded mesoporous silicon nanoparticles on alcoholic hepatitis through mTOR-mediated autophagy pathway, Biomedical microdevices 24(4) (2022) 37.

[7] B. Li, W. Zhou, X. Wang, W. Zhong, Q. Zheng, Y. Chen, P. Yue, Bilayer-functionalized mesoporous silica nanoparticles for overcoming multiple barriers of mucus clearance, intestinal epithelium and P-glycoprotein efflux, Materials & Design 240 (2024) 112820.

[8] W. Deng, H. Wang, B. Wu, X. Zhang, Selenium-layered nanoparticles serving for oral delivery of phytomedicines with hypoglycemic activity to synergistically potentiate the antidiabetic effect, Acta pharmaceutica Sinica. B 9(1) (2019) 74-86.

[9] J. Guo, X. Xing, N. Lv, J. Zhao, Y. Liu, H. Gong, Y. Du, Q. Lu, Z. Dong, Therapy for myocardial infarction: In vitro and in vivo evaluation of puerarin-prodrug and tanshinone co-loaded lipid nanoparticulate system, Biomedicine & pharmacotherapy = Biomedecine & pharmacotherapie 120 (2019) 109480.
